# Monoclonal Antibodies for Rift Valley Fever Virus Nucleocapsid: Application in IgG/IgM ELISA for Sero-Diagnosis

**DOI:** 10.3390/pathogens13070582

**Published:** 2024-07-13

**Authors:** Jiansheng Huang, Ferdinard Adungo, Samson Limbaso Konongoi, Shingo Inoue, Lin Zhan, Rosemary Sang, Salame Ashur, Allan ole Kwallah, Matilu Mwau, Kouichi Morita, Fuxun Yu

**Affiliations:** 1 School of Medicine, Guizhou University, Guiyang 550002, China; 18084642208@163.com; 2 Kenya Medical Research Institute, Nairobi 54840-00200, Kenya; ferdinard.adungo@gmail.com (F.A.); limbaso.konongoi@usamru-k.org (S.L.K.); rosemary.sang@usamru-k.org (R.S.); salameashur@gmail.com (S.A.); akwallah@gmail.com (A.o.K.); matilu.mwau@gmail.com (M.M.); 3 Department of Virology, Institute of Tropical Medicine, Nagasaki University, 1-12-4, Sakamoto, Nagasaki 852-8523, Japan; samakirice2012@gmail.com (S.I.); moritak@nagasaki-u.ac.jp (K.M.); 4 NHC Key Laboratory of Pulmonary Immunological Diseases, Guizhou Provincial People’s Hospital, Guiyang 550002, China; zhanlin300@hotmail.com; 5 Guizhou Provincial People’s Hospital, Guizhou University, Guiyang 550002, China

**Keywords:** Rift Valley fever virus, nucleocapsid protein, monoclonal antibody, IgG ELISA, IgM capture ELISA

## Abstract

Introduction: Rift Valley fever virus (RVFV) belonging to the Phenuiviridae family is responsible for a zoonotic disease called Rift Valley fever (RVF). Currently, RVFV has spread from Africa to Asia, and due to its ability to cause high mortality rates, it has significantly impacted human health and economic development in many societies. Highly specific and sensitive systems for sero-diagnosis of RVFV infection are needed for clinical use. Method: BALB/c mice were immunized with recombinant RVFV nucleocapsid (rRVFV-N) protein and the spleen cells fused with SP2/0 myeloma cells to create hybridoma cell lines. The secreted monoclonal antibodies (MAbs) were purified and characterized. Enzyme-linked immunosorbent assay (ELISA) systems for the detection of IgG and IgM using the new MAbs were established and evaluated. Serum samples from 96 volunteers and 93 patients of suspected RVF from Kenya were tested compared with the ELISA systems based on inactivated viruses and the rabbit polyclonal antibody. Result: Three monoclonal antibodies against rRVFV-N protein were established. The performance of the MAb-based sandwich IgG ELISA and the IgM capture ELISA perfectly matched the ELISA systems using the inactivated virus or the polyclonal antibody. Conclusions: Recombinant RVFV-N protein-specific MAbs were developed and they offer useful tools for RVFV studies. The MAb-based ELISA systems for detecting IgG and IgM offer safe and useful options for diagnosing RVFV infections in humans.

## 1. Introduction

Rift Valley fever (RVF) is a zoonosis predominantly transmitted through a mosquito bite or contact with infected body fluids. The clinical manifestations of RFV include nonspecific symptoms similar to influenza, ocular and central nervous system complications, multiple organ failure, and potentially fatal outcomes [1,2,3]. Additionally, RVF is linked to miscarriages, stillbirths, and congenital infections in humans [4].

To date, no licensed human RVF vaccine or treatment exists, leaving millions of people in endemic areas at a greater risk of infection and devastating disease consequences [5]. The World Health Organization (WHO) estimates that 200,000 human cases occur annually, with at least a 1% fatality rate. RVF infections may lead to about 1.98 DALYs per 1000 populations, threatening food security and straining healthcare systems especially in resource-limited settings [6].

First identified in the 1930s, RVF has caused large-scale outbreaks beginning in the 1950s [5]. South Africa reported 110 human cases and 7 fatalities between 1974 and 1975 [6]; Egypt experienced over 200,000 infections and 598 deaths due to RVF between 1977 and 1979 [7]. In 1987, Mauritania reported an outbreak resulting in 220 deaths [8]. Additionally, in 2000, Saudi Arabia and Yemen reported large-scale outbreaks [9,10]. The epidemic in Kenya that occurred between 2006 and 2007 impacted 18 regions across six provinces, exhibiting extremely high mortality rates and up to 180,000 infected individuals in the most severely affected areas [11]. Serum collection from local residents revealed an incidence rate of RVF as high as 13% in these areas [11]. Smaller-scale outbreaks were also documented in Uganda from 2017 to 2018 [12], posing a significant threat to human health and socio-economic development in affected area.

RVFV belongs to the order Bunyavirales, family Phenuiviridae, and genus Phlebovirus. It has a single-stranded RNA genome enclosed by the viral envelope, comprising of three segments: Large, Medium, and Small [13]. The Large segment codes for the t RNA polymerase, while the Medium segment codes for the glycoproteins Gn and Gc, the non-structural protein NSm, P78, P14, and P13. The Small segment encodes the nucleocapsid N protein and the NSs protein, which are associated with viral virulence [14,15,16]. Notably, the nucleocapsid N protein is conserved across different strains of the RVF virus, offering a significant immunological advantage within the Phenuiviridae family [17,18]. The L and N are vital for viral replication and transcription, with potential to induce human T cell responses. At early/viremic stages of RVFV infection, the N protein is usually expressed at high levels making it an important target for the development of diagnostics and vaccines.

Although RT-qPCR has been adeptly utilized in clinical settings to detect viremia in RVF patients [19,20,21], assess virus titers [22], and infer the presence of RVF genomes and antigens (L, M, S) [23], isothermal amplification techniques like loop-mediated isothermal amplification (LAMP) and recombinase polymerase amplification (RPA) have demonstrated comparable sensitivity without necessitating complex instruments, thus facilitating field deployment [24,25]. However, these methods encounter challenges such as the short duration of viremia (within 7 days after infection) and a high dependency on specialized equipment and highly trained professionals [26,27]. Serological diagnosis, identifying IgM antibodies present from the fourth day of infection and IgG antibodies persisting years after the eighth day of infection, emerge as critical diagnostic tools for RVF [28,29]. Our prior research has established a sandwich ELISA detection system coated with rabbit polyclonal antibody against RVFV, showing 100% concordance compared to conventional ELISA systems based on inactivated RVFV [30]. Nevertheless, compared with polyclonal antibodies, monoclonal antibodies have the advantages of less batch-to-batch variation and higher specificity. Thus, further validation and refinement of these detection methods are needed.

In this study, MAbs against rRVFV-N protein were developed by fusing spleen cells from immunized BALB/c mice with mouse myeloma SP2/0 cells. The MAbs were used to make a sandwich IgG ELISA system and IgM capture ELISA system for human serum. The efficacy of these newly established ELISA systems was evaluated by comparing with those from WHO-recommended ELISA systems using RVF inactivated virus or our previously established ELISA systems using rabbit polyclonal antibody against RVFV [30].

## 2. Materials and Methods

### 2.1. Human Serum

The Kenya Medical Research Institute provided 193 human serum samples for this study comprising 2 positives, 2 negatives, 96 samples from volunteers, and 93 samples from RVF suspected patients. The positive and negative samples were used as controls to monitor the ELISA systems. Volunteer samples collected in 2013 during an RVF surveillance study in Kenya with some IgG positive results were used to evaluate the IgG ELISA systems. RVF-suspected patient samples that were collected during the 2006–2007 RVF outbreak in Kenya with IgM positive results were used to evaluate the IgM ELISA systems.

### 2.2. Recombinant rRVFV-N Protein Expression and Purification

The rRVFV-N protein was expressed and purified as previously reported [30]. Briefly, the specific N protein gene sequence was cloned into pQE30 vector (Qiagen, Hilden, Germany). The recombinant plasmids were inserted to *E. coli* XL-1 for large-scale propagation. Isopropyl β-D-thiogalactoside (IPTG) was used to induce the expression of rRVFV-N. The *E. coli* was harvested by centrifugation and the resultant cell suspension sonicated following a series of freeze-thawing steps. The final cell suspension was centrifuged at 20,000× *g* for 30 min and applied to the Talon^TM^ IMAC column for purification. The rRVFV-N was analyzed by sodium dodecyl sulfate-polyacrylamide gel electrophoresis (SDS-PAGE).

### 2.3. Mouse Immunization Schedule

Mouse immunization was segmented into three phases, spanning a total of 44 days. Phase 1: 100 mg (2000 mg/kg body weight) of rRVFV-N proteinin 0.5 mL was mixed with an equivalent volume of Freund’s complete adjuvant (MP Biochemicals, Santa Ana, CA, USA) and intraperitoneally administered to two male BALB/c mice aged 6 weeks (Chongqing Tengxin Bill Experimental Animal Sales Co., LTD, Chongqing, China). Phase 2: 2 weeks later, the mice received a booster injection with the same proportion of Freund’s incomplete adjuvant and rRVFV-N protein, followed by another booster on day 28 to enhance immunization. Ten days after immunization, blood samples were collected to measure the antibody titers using indirect ELISA. Phase 3: administering 100 mg of rRVFV-N protein intraperitoneally on days 42, 43, and 44. On day 45, the mouse was euthanized, blood collected, and the spleens harvested for cell fusion.

### 2.4. Monoclonal Antibody Production

After immunization, hybridoma cells were generated using spleen cells and SP2/0 myeloma cells (from previous experiments [31]). Spleen cells used were 1 × 10^8^ and the SP2/0 cells used were 2 × 10^7^, which give a cell ratio of 5:1. Polyethylene glycol 1500 (Roche, Indianapolis, IN, USA) is biologically evaluated for high fusion efficiency and was used to catalyze the cell fusion process. The resultant hybridoma cells were cultured using a selective medium containing hypoxanthine aminopterin thymidine (HAT) in 10% fetal bovine serum RPMI 1640 (Gibco, Grand Island, NY, USA). HAT medium selectively kills unfused myeloma cells and eliminates myeloma–myeloma hybridomas that lack hypoxanthine-guanine phosphoribosyl transferase (HPRTase) and thymidine kinase (TK) enzymes needed for the breakdown of aminopterin, which blocks DNA synthesis. The supernatants were screened for cells capable of secreting antibodies against rRVFV-N protein using indirect ELISA. Positive clones were purified by limited dilution employing across three or more rounds. Briefly, positive hybridoma were diluted in RPMI 1640 medium and propagated in 96-well microplates to achieve a distribution of one cell per well. After culturing for 7 days, antibody-secreting clones were confirmed using indirect ELISA. The above steps were repeated three times to purify positive clone capable of secreting anti-rRVFV N protein MAbs. Positive clones were finally transferred to Hybridoma SFM medium (Gibco, NY, USA) and cultured for 7 days for large-scale antibody production or re-injected intraperitoneally into BALB/c mice for ascites fluid collection. The monoclonal antibodies were purified using HiTrap Protein G HP antibody purification columns (GE Healthcare, Uppsala, Sweden), according to the manufacturer’s instructions.

### 2.5. Screening Positive Cell Lines via Indirect IgG ELISA

To screen for positive hybridoma cell lines, we adapted the previously established IgG ELISA screening protocol [31]. Briefly, 50 ng/well of purified rRVFV-N protein was coated overnight in a 96-well microplate at 4 °C, then blocked with PBS-T containing 5% skim milk at room temperature for an hour. Then, 100 μL of hybridoma cell supernatant, along with diluted pre-immunized and post-immunized mouse serum, was added to each well as control. The microplate was incubated for 1 h at room temperature and washed with PBS-T three times before adding 100 μL of diluted horseradish peroxidase-labeled (HRP) goat anti-mouse IgG (American Qualex, San Clemente, CA, USA). The working concentration of the HRP-labelled goat anti-mouse was 1:10,000. Following another hour of incubation at room temperature and a washing step, 100 μL of ABTS substrate solution [2,2′ azinobis (3-ethylbenzothiazoline sulfonic acid)] (Roche Diagnostics, Mannheim, Germany) was added per well and incubated in the dark for 30 min. The optical density readout, which was two times higher than the OD of the negative control, was deemed positive.

### 2.6. Immunofluorescence Analysis

Vero E6 cells (ATCC USA) were cultured on cell slides (NEST, Shanghai, China) for 4 days before being inoculated with 80 μL RVFV (Smithburn strain GenBank: OP146109.1) or PBS solution (mock infection). Three days later, the slides were then fixed with 4% paraformaldehyde (Biosharp, Wuhan, China) for 30 min. Cell permeabilization was carried out using 1% Triton X-100 solution. The slides were blocked using 5% BSA for 1 h, and mouse ascites diluted 1:10,000 were added and incubated overnight at 4 °C. Next, goat anti-mouse IgG conjugated with fluorescein (FITC) (Bethyl Laboratories Inc., Montgomery, TX, USA) was diluted at 1:50 and applied to each well and incubated in the dark for 1 h under room temperature. The slides were washed three times with PBS, before each reagent addition step. The Olympus IX73 immunofluorescence microscope was used to capture the final images. Immunofluorescence assay was used to check the MAb’s reaction to SFTS virus, H1N1 influenza virus, respiratory syncytial virus (RSV), and EV71 virus infected cells. Immunofluorescence results of the three MAb strains were quantified using Fiji Image J 1.54f and GraphPad Prism 9.5.

### 2.7. Western Blot Experiments

Western blotting was used to check the reactivity of the monoclonal antibodies to the purified rRVFV-N protein. It was carried out following the previously described protocol with minor modifications [30]. A one-step PAGE gel rapid preparation kit (YAZY, Shanghai, China) was used to prepare 15% gel. The purified rRVFV-N protein and a protein molecular size marker (PageRulerTM Prepared Protein Ladder, Thermo Scientific Denmark) samples were mixed with loading buffer and applied separately for electrophoresis. The separated proteins were then immobilized on a polyvinylidene fluoride (PVDF) membrane using a wet transfer system (Immobilion, Millipore, Massachusetts, USA). Blocking was achieved by immersing the PVDF membrane in 5% skim milk at room temperature for 1 h, washed three times with TBST, and reacted with mouse hybridoma ascites diluted at 1:6000 or unimmunized mice serum diluted 1:200 (as negative control). After incubating overnight at 4 °C, the PVDF membrane was washed three times using TBST buffer before adding HRP-labeled goat anti-mouse secondary antibody (BioPM, Wuhan, China) diluted at 1:10,000. To allow for binding, an incubation step of 2 h at 4 °C was performed. Following a final washing step with TBST three times, the immobilized proteins were visualized using a metal-enhanced DAB substrate solution (Solarbio, Beijing, China) according to the manufacturer’s instructions. 

### 2.8. MAb Subtyping 

Subtyping of three selected monoclonal antibodies was carried out using a mouse MAb typing kit (Roche, New York, NY, USA) following instructions from the manufacturer.

### 2.9. ELISA Procedures

To assess the usefulness of the newly developed MAbs on sero-diagnosing human RVF infection, IgG antibody sandwich ELISA and IgM capture ELISA using the newly developed MAbs were established. Samples were checked in parallel using ELISA coated with rabbit polyclonal antibody against RVFV or WHO-recognized laboratory routine ELISA systems using inactivated Rift Valley fever virus. The common ELISA procedures involved coating a 96-well immunoassay plate (Thermo Scientific, Denmark) with 100 μL per well, washing the plate three times with 0.01 M PBS+0.1% Tween 20 (PBS-T) after each reagent reaction. Coating was performed overnight at 4 °C with 0.01 M PBS, and all serum samples and reagents were diluted in PBS-T containing 5% skim milk (Difco, Detroit, MI, USA). All incubation was carried out at 37 °C for 1 h, except H_2_O_2_-ABTS substrate (Kirkegaard & Perry, Gaithersburg, MD, USA), which was applied at 100 μL/well, then incubated in the dark at 37 °C for 30 min. In each ELISA experiment, two positives and two negatives were included as controls. Duplicate testing of each serum was carried out and the OD values at 410 nm wavelength recorded. All samples yielding mean OD values 2 times higher than the mean OD values of the negative control were regarded as positive. 

Checkerboard titration using two positive and two negative controls was used to determine the optimal dilutions of the human serum and monoclonal antibodies used for coating the ELISA microplates. 

### 2.10. IgG Sandwich ELISA Based on MAbs against rRVFV-N Protein

An IgG sandwich system that employs MAbs against RVFV as a coating antibody was developed. The 3 MAbs generated similar results when used separately or mixed thus MAb 5F11 was used in later experiments. The prepared MAb 5F11 was diluted 1:10,000 and coated into all wells at 4 °C overnight. After blocking for 1 h with 5% skim milk, 30 ng/well of rRVFV-N protein (positive antigen), or recombinant severe fever with thrombocytopenia syndrome virus nucleocapsid protein (rSFTSV-N) (negative antigen) [32] in PBS was then reacted with the coating MAbs. After reaction, serum diluted at 1:100 concentration was added to four wells: two of rRVFV-N protein wells and two of rSFTSV-N protein wells. The plate was then reacted with diluted HRP-conjugated goat anti-human IgG (American Qualex, CA, USA) at a concentration of 1:30,000 for 1 h at 37 °C. Finally, ABTS substrate was introduced, and the wells were incubated in darkness to facilitate color development.

### 2.11. IgM Capture ELISA System Based on MAbs against rRVFV-N Protein

An IgM capture ELISA detection system was developed using newly generated MAbs. To test each serum sample, four microplate wells were coated with 1:500 goat anti-human IgM (Kirkegaard&Perry Laboratories) overnight at 4 °C. Following a blocking step using 5% skim milk, 1:100 dilution of serum from a suspected patient was added to the four wells. After reaction, 50 ng/well of rRVFV-N protein (positive antigen) was added to two wells, while the other two wells were added with 50 ng of rSFTSV-N protein (negative antigen). Then 1:10,000 MAbs against rRVFV-N protein was added to all wells before reaction with HRP-conjugated anti-mouse IgG (American Qualex, CA, USA) using a 1:10,000 concentration. Finally, ABTS substrate was added before measuring the OD values.

### 2.12. ELISA Systems Used for Comparison

To assess the efficacy of the newly developed ELISA systems, samples were checked in parallel using the WHO-recommended IgG sandwich ELISA, IgM capture systems using inactivated Rift Valley fever virus as an antigen, and the rabbit polyclonal antibody-based IgG sandwich ELISA, IgM capture ELISA systems using rRFV-N protein as antigen. These tests were carried out as described in our previous study [30]. 

### 2.13. Statistical Analysis

The comparison between two ELISA assays were checked for consistency by using the Kappa coefficient.

## 3. Result

### 3.1. Development and Preliminary Characterization of RVF Monoclonal Antibodies

**Figure 1:**
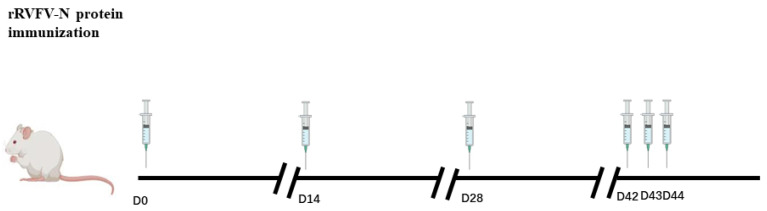
BALB/c mice immune schedule.

After immunization (according to the immune scheme depicted in Figure 1), spleen cells from BALB/c mice were harvested and chemically fused with SP2/0 cells to produce hybridoma cells. Following three rounds of limited dilution culture and screening, three hybridoma cell clones steadily secreting MAbs against rRVFV-N protein were obtained. The three MAbs were named as 3D11, 4H3, and 5F11.

**Figure 2:**
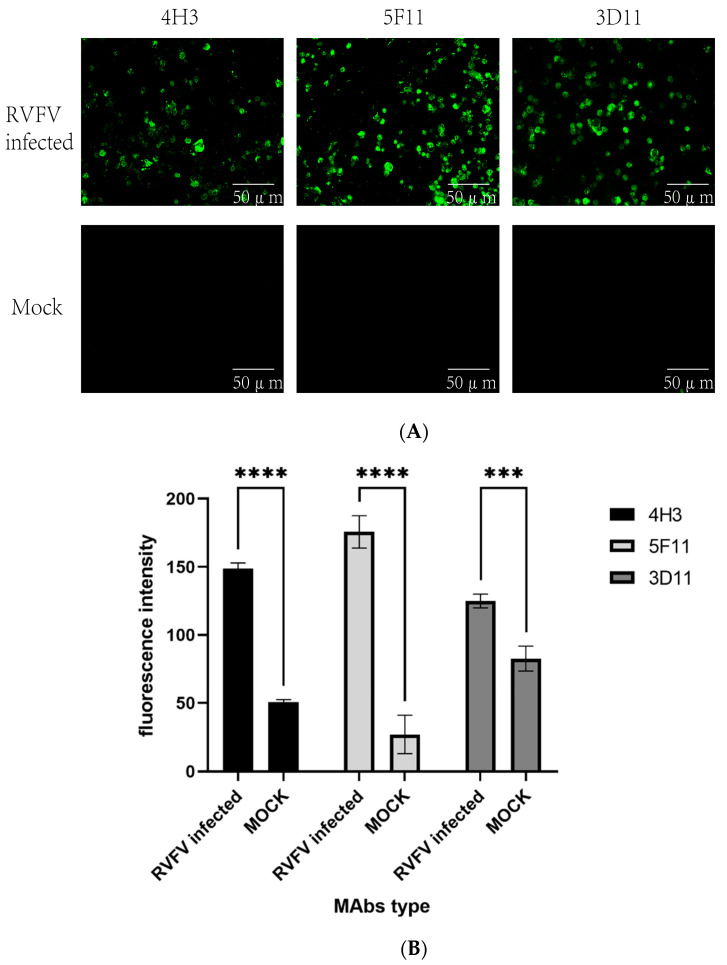
(**A**) Immunofluorescence analysis of the three MAbs’ reaction with RVFV or mock infected Vero-E6 cells. (**B**) Fluorescence intensity of three strains of MAbs. (The significance of the difference was indicated using ‘****’ (*p* < 0.0001) and ‘***’ (*p* = 0.0002)).

**Figure 3:**
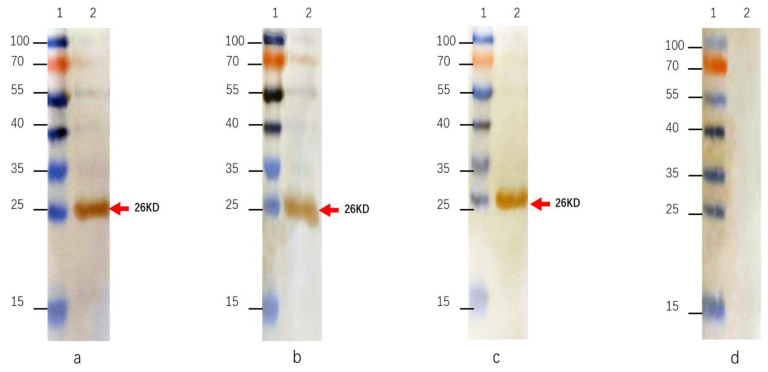
Western blotting of MAbs against rRVFV-N protein. (**a**): MAb 4H3; (**b**): MAb 5F11; (**c**): MAb 3D11; (**d**): negative control. Lane 1: protein size marker; Lane 2: purified rRVFV-N protein.

**Figure 4:**
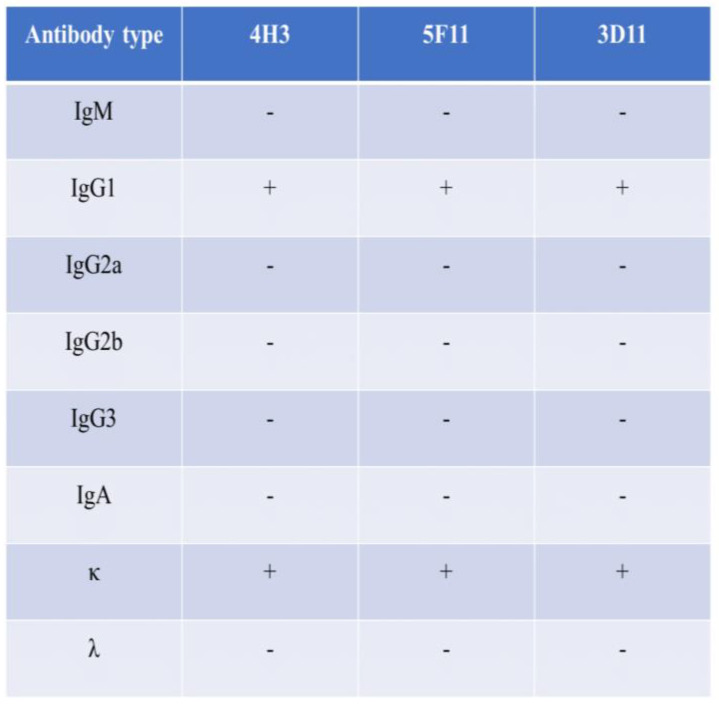
Antibody subclasses. The subclasses of the three MAbs belong to IgG1 and κ chain.

The indirect immunofluorescence assay demonstrated that RVFV infected Vero E6 cells exhibited fluorescence when reacted with the three MAbs, whereas no fluorescence could be detected in uninfected cells (Figure 2). The MAbs do not react with SFTSV, H1N1, RSV, and EV71 virus. Further characterization of these antibodies through Western blot showed that all three could bind to the rRVFV-N protein (Figure 3). Antibody subtyping showed that all strains have an IgG1 heavy chain with a kappa light chain (Figure 4).

Two mice were immunized with rRVFV-N protein mixed with an equivalent volume of Freund’s complete adjuvant at day 0, with rRVFV-N protein mixed with a proportional equal volume of Freund’s incomplete adjuvant at day 14 and day 28, with rRVFV-N protein at day 42, 43, and 44.

### 3.2. Evaluation of MAb-Based IgG ELISA System

To assess the utility of newly generated monoclonal antibodies on sero-diagnosis, sandwich IgG ELISA system was developed. Serum samples from 96 human volunteers were tested; MAb-based IgG ELISA identified 11 positives and 85 negatives. These results were 100% concordant with those from the IgG capture system using inactivated virus and rabbit polyclonal antibody-based sandwich IgG ELISA and the kappa coefficients between them are all 1 (Table 1 and Table 2), indicating the MAbs’ usefulness in the IgG ELISA system.

### 3.3. Evaluation of MAb-Based IgM Capture ELISA System

Of the 93 serum samples from suspected Rift Valley fever patients tested, 42 were IgM positive. This result matched 100% with the IgM capture system based on inactivated viruses and rabbit polyclonal antibody and the kappa coefficients between them are all 1 (Table 3 and Table 4), indicating that the newly developed MAbs could be used in IgM capture ELISA system.

## 4. Discussion

Rift Valley fever is a viral zoonosis mainly transmitted via bites of infected mosquitoes, capable of causing human hemorrhagic fever, hepatitis, encephalitis, and retinal vascitis. The mortality rate for severe infections can reach up to 50% [33], with evidence indicating spread of the virus from Africa to the Arabian Peninsula and the Middle East [9,10]. However, no vaccines or drugs against Rift Valley fever are currently approved for human use worldwide [34], making early prevention and timely, effective diagnosis crucial in combating the disease. Zaki et al. developed a method for producing monoclonal antibodies to RVFV and utilized these antibodies in an IgG ELISA assay to detect the RVFV virus in cell cultures [18]. Fafetine et al. described a method for preparing and identifying a monoclonal antibody against RVFV recombinant nucleocapsid protein [35]. Catherine Cetre-Sossah et al. developed a lateral flow immunochromatographic strip using a monoclonal antibody against RVFV nucleocapsid protein to detect serum samples from animals [36]. These studies did not apply the MAbs for IgG and IgM capture ELISA for human serum, thus underscoring the effectiveness of monoclonal antibodies against the RVF nucleocapsid protein for sero-diagnosis in human infection. It is crucial to establish affordable, safe, and accurate RVF diagnosis methods for human clinical application.

In this study, hybridoma cells were generated by fusing immunized BALB/c mouse spleen cells (as depicted in Figure 1) with SP2/0 myeloma cells following three rounds of immunization. Three monoclonal antibodies against RVFV, named 3D11, 4H3, and 5F11, were obtained. Preliminary characterization of these antibodies revealed that the three antibodies could specifically bind to RVFV-infected but not mock Vero E6 cells in an immunofluorescence assay (Figure 2). Additionally, Western blot analysis revealed that all three could bind to the rRVFV-N protein, resulting in visible bands (Figure 3). Antibody isotyping identified all three antibodies as IgG1 heavy chain and κ light chains (Figure 4) that may be because of the high dose of recombinant antigen used for immunization so this type of MAb was dominant. To further assess the diagnostic capability of these MAbs, sandwich IgG ELISA and IgM capture ELISA systems were developed utilizing the MAb as the core component. These systems were used to detect serum samples from 96 volunteers and 93 suspected RVF patients and the results were comparable to those of the WHO-recommended ELISA systems using inactivated viruses and our previously reported rabbit polyclonal antibody and rRVFV-N protein based-ELISA systems. Of the 96 serum samples tested by the IgG sandwich ELISA system, 11 were positive and 85 were negative. Meanwhile, of the 93 suspected RVF patient serum samples tested by the IgM capture ELISA system, 42 were positive and 51 were negative. These results achieved a 100% match with the other two ELISA systems. MAbs offer a more stable and more specific source of antibody, thus improving diagnostic accuracy and reliability compared to polyclonal antibodies. It is a useful tool for RFV diagnosis and for supporting future research. The MAbs developed in this study could also be used for antigen-detection ELISA and immunochromatography tests for early and bedside diagnosis of RVFV infections in humans and animals.

Compared with the molecular biology diagnostic methods currently utilized for the detection of RVF virus during the viremic stage (within 7 days of infection), the antibody-detection methods developed in this study could not only reduce the dependence on specialized equipment and skilled personnel but are also useful for testing patients out of the viremia stage, thus extending the detection window. Moreover, the detection approach employed here, using sandwich IgG and IgM capture systems, does not necessitate high-level biosafety facilities or direct contact with the virus, rendering it suitable for field deployment in epidemiological studies and application in resource-limited countries. Next, scale-up and standardization of the systems are needed for commercializing the kits.

Nevertheless, this study faced several limitations, including scarcity of remaining serum and the inability to perform neutralization tests. The serum samples analyzed were not validated using alternative testing methods, such as molecular biology techniques, and the sample size was relatively small. Prior to a broader application, it is essential to validate these methods with serum samples from a wider array of countries and regions. The newly developed systems need to be evaluated by comparison with neutralization tests or other molecular methods which could confirm the infection.

## 5. Conclusions

Three monoclonal antibodies against RVF virus were developed. A sandwich IgG ELISA and an IgM capture ELISA system for human serum were established using the MAbs. The MAb-based ELISA systems work well for human RVF clinical diagnosis. The MAbs offer useful tools for RVF diagnosis and for further research applications.

## Figures and Tables

**Table 1:** Comparation of MAb-based IgG sandwich ELISA with inactivated virus-based IgG sandwich ELISA for human serum.

MAb-Based IgG Sandwich ELISA	Inactivated Virus-Based IgG ELISA		Total
	Positive	Negative	
Positive	11	0	11
Negative	0	85	85
Total	11	85	96
Concordance ^a^: 100%	Sensitivity ^b^: 100% Specificity ^c^: 100%	PPV ^d^: 100%	NPV ^e^: 100%

^a^ (No. of samples positive by both methods + No. of samples negative by both methods)/total number of samples × 100; ^b^ True positive/(true positive + false negative) × 100; ^c^ True negative/(true negative + false positive) × 100; ^d^ True positive/(true positive + false positive) × 100; ^e^ True negative/(true negative + false negative) × 100.

**Table 2:** Comparation of MAb-based IgG sandwich ELISA with rabbit polyclonal antibody-based IgG ELISA for human serum.

MAb-Based IgG Sandwich ELISA	Rabbit Polyclonal Antibody-Based RVFV IgG ELISA		Total
	Positive	Negative	
Positive	11	0	11
Negative	0	85	85
Total	11	85	96
Concordance: 100%	Sensitivity: 100% Specificity: 100%	PPV: 100% NPV: 100%	

**Table 3:** Comparation of MAb-based IgM capture ELISA with inactivated virus-based IgM capture ELISA for human serum.

MAb-Based IgM Sandwich ELISA	Inactivated Virus-Based IgM ELISA		Total
	Positive	Negative	
Positive	42	0	42
Negative	0	51	51
Total	42	51	93
Concordance: 100%	Sensitivity: 100% Specificity: 100%	PPV: 100% NPV: 100%	

**Table 4:** Comparation of MAb-based IgM capture ELISA with rabbit polyclonal antibody-based IgM capture ELISA for human serum.

MAb-Based IgM Capture ELISA	Rabbit Polyclonal Antibody-Based IgM Capture ELISA		Total
	Positive	Negative	
Positive	42	0	42
Negative	0	51	51
Total	42	51	93
Concordance:100%	Sensitivity: 100% Specificity: 100%	PPV:100% NPV: 100%	

## Data Availability

The original contributions presented in the study are included in the article; further inquiries can be directed to the corresponding author.

## Abbreviations

RVFV, Rift Valley fever virus; MAbs, Monoclonal antibodies; RT-PCR, Reverse transcription polymerase chain reaction; LAMP, Loop-mediated isothermal amplification assay; RPA, Recombinase Polymerase Amplification; ELISA, Enzyme-linked immunosorbent assay; IgG, Immunoglobulin G; IgM, Immunoglobulin M; OD, Optical density; rRVFV-N, Recombinant RVFV-N; RVFV, Rift Valley fever virus; HAT, Hypoxanthine aminopterin thymidine; DPBS, Dulbecco’s Phosphate-Buffered Saline; PVDF, Polyvinylidene fluoride; TBST, Tris-Buffered Saline and Tween 20; PBST, 0.01 M PBS with 0.1% Tween 20; FBS, Fetal bovine serum; HRP, Horseradish-peroxidase-conjugated; FITC, Fluoresceine isothiocyanate.
